# Thermodynamic Conditions for Consolidation of Dissimilar Materials in Bimetal and Functional Graded Structures

**DOI:** 10.3390/ma15030825

**Published:** 2022-01-21

**Authors:** Alexander Khaimovich, Yaroslav Erisov, Igor Shishkovsky

**Affiliations:** 1 Engine Production Technology Department, Samara National Research University, 34 Moskovskoye Shosse, 443086 Samara, Russia; berill_samara@bk.ru; 2 Metal Forming Department, Samara National Research University, 34 Moskovskoye Shosse, 443086 Samara, Russia; 3 Samara Federal Research Center, Russian Academy of Sciences, 3A Studencheskiy Pereulok, 443001 Samara, Russia; 4 Center for Design, Manufacturing and Materials, Skolkovo Institute of Science and Technology (Skoltech), Bolshoy Boulevard 30, bld. 1, 121205 Moscow, Russia; shishkowsky@gmail.com

**Keywords:** functional graded intermetallic structures, functional bimetallic materials, thermodynamical approach, adhesive consolidation criteria, 316L, AlSi10 Mg, computational thermodynamics

## Abstract

Functional Graded Structures and Functional Graded parts, made using dissimilar materials, are designed to provide specific properties to the final product. One of the most promising methods for manufacturing 3D Functional Graded objects is 3D laser cladding and/or direct energy deposition. However, the construction of graded and especially layered graded structures in the process of joining materials with different thermophysical properties under certain conditions is accompanied by the formation of cracks along the phase boundaries, which are a consequence of residual stresses and/or chemical segregations. The conditions for phase consolidation are macroscopic balancing of residual stresses in the region of the interface. In a broader sense, in the field of the interface, it is necessary to consider the thermodynamic equilibrium of the phases in connection with mechanical equilibrium. In this regard, the article proposed criteria for the thermodynamic affinity of phases in the area of the Functional Graded Structures interface, including the coefficients of thermal expansion and isobaric and isochoric heat capacities of the phases. Examples of cracking and the use of the obtained criteria are provided.

## 1. Introduction

Functional Graded Materials (FGMs) are in demand in changing operating conditions, which, depending on the design of the parts made from them, require corresponding variability to the characteristics of the materials [1]. The extreme operating conditions include aerospace structures [2], nuclear power, and other applications that require the performance of parts at radically different temperatures [3]. Material properties such as corrosion resistance, strength, toughness, wear resistance at low weight, and reasonable cost are rarely, if ever, found in a single material [4,5]. Therefore, technologies are required to ensure the connection of dissimilar materials or a functional gradient of properties for two or more materials [1,6]. Sufficiently complete classification of FGMs was suggested in [7].

With a sharp difference between the chemical composition of the FGM phases, one can speak of functional bimetallic materials (FBMs). As a means of obtaining materials for a thermal barrier, FBMs were proposed in 1984 in Japan [8]. FBMs are advanced materials that can achieve a transition gradient or mostly graded transition from one material to another for a variety of materials [9]. In general, Functional Graded Structures (FGSs) and Functional Graded (FG) parts made using dissimilar materials are designed to provide specific properties to the final product. Mix of dissimilar materials can create different types of intermetallic compounds at the interface, which lead to properties that are difficult to predict [10]. The fabrication of 3D FG objects by 3D laser cladding and/or direct energy deposition (DED) is one of the most promising methods for solving various industrial problems [11]. Laminated metal matrix composites are intermediate between FGMs and FBMs.

**Figure 1:**
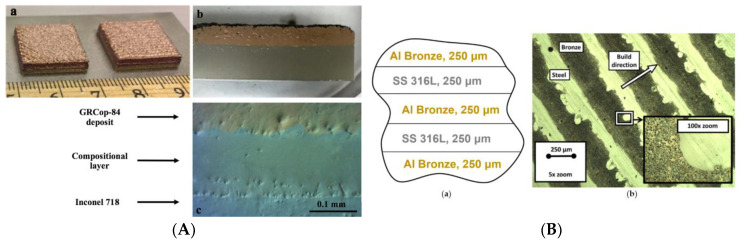
Optical image of the compositional interface: (**A**) as fabricated bimetallic structures of Inconel 718 and GRCop-84 [12]; (**B**) the resulting graded structure from SS316L—Al Bronze [13] (Reprinted with permission from Ref. [13] Elsevier 2021).

Figure 1 shows bimetallic samples [12] of Inconel 718-copper alloy obtained by the LENS^TM^ technology and laminated FGM from SS316L—Al Bronze [13] obtained by DED method.

The joining of dissimilar materials is usually difficult because of the significant difference in thermophysical properties between two materials, along with the possibility of the formation of brittle intermetallic phases [14]. After crystallizing and further cooling of the melt volume in DED of bimetallic Ti6Al4V + Al12Si structures via additive manufacturing, the interfacial area can experience a significant thermal mismatch caused by residual stresses due to the difference in the coefficient of thermal expansion [15].

To overcome this problem, researchers have developed a method for producing a composite-gradient transition zone as an interfacial area instead of directly fusing layers of dissimilar materials or fabricating bimetallic structures. Bimetallic structures such as Al/Ti6Al4V, SS410/Stellite™, CuSn/18Ni300, and SS316L/CuSn10 were fabricated using additive manufacturing laser technologies [16,17,18,19]. The results show that each metal retained its own properties while still having good bond strength between the two metal materials due to the dispersed interface.

The formation of gradient and especially layered graded structures under certain conditions is accompanied by the formation of cracks. In [20], when incrementally graded to SS304L with Inconel 625 by DED technology, cracks of hundreds of microns were found in the area of the second phase precipitation (in the area containing approximately 79 wt.% SS304L and 21 wt.% Inconel 625). The researchers did not find any macroscopic compositional segregation to indicate the reason for such cracks. While austenitic stainless steels that solidify directly into the austenite phase are known to be susceptible to solidification cracking during welding, stainless steel 304L solidifies from primary dendritic ferrite to austenite, and is therefore not susceptible solidification cracking. This indicates that the cracks found are not due to intrinsic solidification cracking in the stainless steel [20].

In [21], during the formation of a graded Ti6Al4V/SS304L structures with an intermediate V section, significant cracks were obtained in the interface due to the precipitation of the σ-FeV phase. The CALPHAD technique was used to calculate the phase equilibria of the Fe-Cr-V and Fe-Ti-V systems. The calculated picture of phase fractions on an isopleth from pure SS304L (Fe-20Cr-9.6Ni) to 50 wt.% SS304L-50 wt.% V calculated at 1123 K showed that after more than 12 wt.% V was added, the only stable phases predicted were the BCC and σ-FeV phases, near and below the crack area. To avoid the formation of Fe-Ti intermetallics that resulted in the cracks at the transition area from 25% Ti-6Al-4V/75% V to 25% SS304L/75% V, a potential alternative pathway would be to grade completely to 100% V before adding SS304L. This would result in a complete transition to V and therefore reduce contact between SS304L and Ti-6Al-4V. So, analyzing the facts, research cannot determine the quantitative reason in phases interface influence that leads to crack appearance [21].

**Figure 2:**
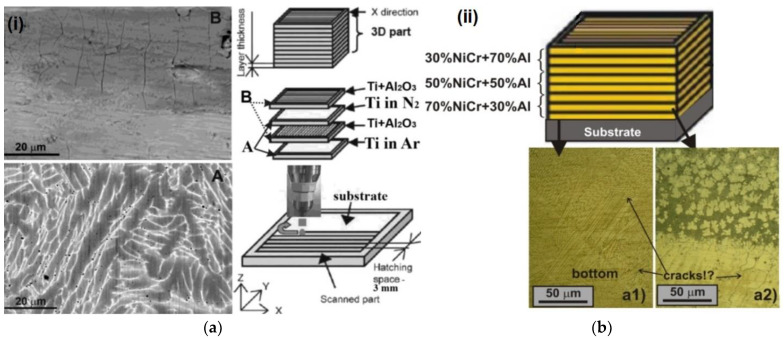
Optical image of the compositional interface: (**a**) cracking in the Ti + Al_2_O_3_ layer when constructing laminated FGM [22] (Reprinted with permission from Ref. [22] Elsevier 2021); (**b**) possible cracks in the interface region of the FGM layer containing 70% NiCr + 30%Al [11].

Figure 2a shows fragments of the microstructure with cracks obtained in the study of the possibility of obtaining a titanium coating containing TiN and aluminum-based ceramics deposited on a Ti6AlV4 substrate after DED in various protective gas environments [22]. During the formation of FGS from a NiCr/Al powder composition by the DED method with a layer-by-layer change in the alloyed composition of the powder from 70% NiCr + 30% Al to 30% NiCr + 70% Al to the upper layer, the probability of cracking in the interface of the lower layer was recorded (Figure 2b) [11].

**Figure 3:**
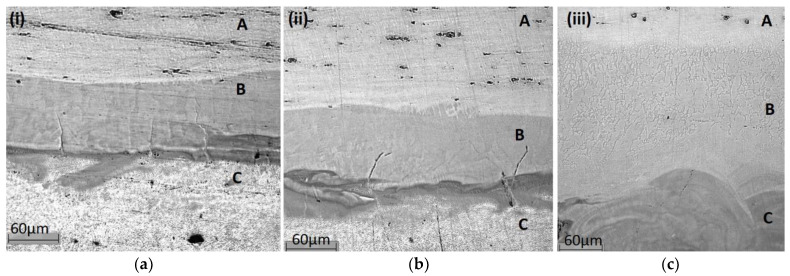
The area of the SS304L/AlSi10Mg interface with cracks when constructing an FBM by the SLM method with a various energy density of the laser beam: (**a**) 105 J/mm^3^, (**b**) 147 J/mm^3^, (**c**) 737 J/mm^3^ (A is the SS304L, B is the thermally stressed interface area, C is the AlSi10Mg) [23].

The authors of this work studied the influence of the main selective laser melting (SLM) parameters of the SS304L-AlSi10Mg bimetal on the features of the formation of the interface region between the layers [23]. As the specific fusion increased, the gradient of the chemical composition in the interface area decreased, which had a significant effect on the crack formation in the thermally stressed area of SS304L-AlSi10Mg (Figure 3).

The formation of cracks when joining dissimilar materials occurs, as a rule, in the area of the interface along the phase boundaries. In any case, the formation of cracks is a consequence of the effect of residual stresses. Residual stresses act over the entire interface, which can lead to the formation of cracks when the thermophysical properties of the phases change during solidification or when the interface is deformed due to changes in the boundary conditions. The conditions for phase consolidation are macroscopic balancing of residual stresses in the interface area. In a broader sense, one can speak not only about mechanical, but also about thermodynamic equilibrium of the interface region. Consolidation in this context is understood as the conditions for maintaining continuity along the boundary and in the near-boundary area of the phase separation.

The CALPHAD (Calculation of Phase Diagrams) technique has made it possible to calculate properties of multicomponent systems using databases of thermodynamic descriptions with models that were assessed from experimental data [24]. The CALPHAD methodology is based on the fact that the phase diagram is a manifestation of the equilibrium thermodynamic properties of the system, which are composed of the properties of the phases that make up the system [25]. In complex systems, computational methods such as CALPHAD are used to simulate thermodynamic properties for each phase and to simulate the behavior of a multicomponent system as a whole. [26].

The purpose of this study was to identify the conditions for the consolidation of phases of a multiphase medium with different thermophysical properties from the conditions of the balance of the thermal and stress-strain states, as well as phase equilibrium in the interface area. The CALPHAD approach was used for the description of chemical segregation behavior into the FBMs after the DED into and near the boundary interfaces.

## 2. Statement of the Problem

Modeling of the bimetallic compound interface was carried out on the basis of the state analysis determined by the thermodynamics of irreversible processes. A similar approach at the macrolevel was used in [27] in relation to a medium consisting of deformable grains. All macroscopic processes in a heterogeneous medium were considered by the methods of continuum mechanics using averaged or macroscopic parameters.

To solve the key problem of finding conditions for the consolidation of a multiphase material from the point of view of thermodynamics, we considered the heat transfer equation at the interface considering the interphase mechanical interaction.

Let us assume that the considered physical model contains a heterogeneous medium in the form of local finite areas (phases), separated by the interface region. In the area of the interface, a change in thermodynamic potentials occurs, caused by different thermophysical properties of the phases.

When formulating the basic assumptions, we use the approach characteristic of multiphase media mechanics [28]:

(i) the dimensions of each phase are many times larger than the characteristic dimensions of the crystal lattice;
(ii) the dimensions of the phase are many times greater than the distances at which the averaged or macroscopic parameters of the deformable medium as a set of its constituent components change significantly.

The first of the assumptions makes it possible to use the classical concepts and equations of the mechanics of continuous single-phase media to describe the processes on the scale of each phase, near the interface. In this case, to describe the thermophysical properties, one can use equations and parameters characteristic of a single-phase state. The second assumption makes it possible to describe macroscopic processes in a heterogeneous medium using averaged or macroscopic parameters.

Considering the accepted assumptions, the mathematical model of the consolidation conditions should contain the equations of energy exchange during interphase interaction in thermal processes of mass transfer and processes associated with elastoplastic deformations. When modeling the state of the medium, it is assumed to consider its functions determined by thermodynamics of nonequilibrium processes that relate stresses and strain rates.

**Figure 4:**
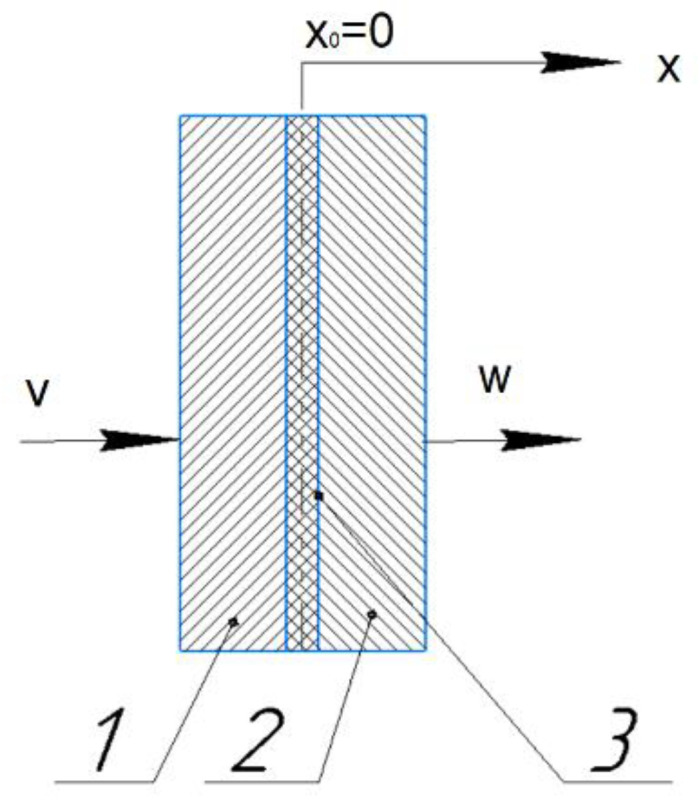
Thermodynamic connection of two phases: 1—phase 1, 2—phase 2, 3—interface area.

Let us define the basic concepts that describe the motion of a deformable medium. Let the velocity field $\vec{v}$ refer to the first phase, and the velocity field $\vec{w}$ to the second phase. We consider the movement of a deformable medium as a thermodynamic connection of two phases that move with the speed $\vec{v}$ and $\vec{w}$ (Figure 4). To connect the velocity fields *v_i_* and *w_i_*, we introduce into consideration a certain coefficient $f_{\rho }$, which is an analytical expression for the Heaviside function:

$$f_{\rho }(x)=\left\{\begin{array}{l} 1,\;x\leq x_{0}=0,\;\exists \frac{\partial f_{\rho }(x_{0}^{-})}{\partial x}\in R^{3},\\ 0,\;x\geq x_{0}=0,\;\exists \frac{\partial f_{\rho }(x_{0}^{+})}{\partial x}\in R^{3}. \end{array}\right.$$

Using the introduced coefficient $f_{\rho }$, we establish a connection between the considered velocity fields:

(1) $$v_{i}^{\Sigma }=(1-f_{\rho })v_{i}+f_{\rho }w_{i}$$

where $v_{i}^{\Sigma }$ is the speed of movement of the consolidated two-phase medium.

## 3. Analytic Solution

Let the phase boundary pass inside the considered volume Ω. Let us denote $\Omega^{v}$, $A^{v}$ as the volume and area of the interface from the side of phase 1 ($x=0^{-}$), respectively, and $\Omega^{w}$, $A^{w}$ as the volume and area from the side of phase 2 ($x=0^{+}$), respectively.

The equation of local mass continuity at the interface can be written in the following form:

(2) $$\frac{d\rho }{dt}+\rho v_{l,l}=-{\left[\rho^{v}f_{\rho }(w_{l}-v_{l})\right]}_{,l},$$

where $\frac{d}{dt}=\frac{\partial }{\partial t}+v_{l}\frac{\partial }{\partial x_{l}}$, $v_{l,l}=\frac{\partial v_{l}}{\partial l}$.

Hereinafter, the operator is used in the subscript for variables $\left(j=\frac{\partial }{\partial x_{j}}\right)$. For tensor components $a_{ij}$ in space $D_{3}$, considering Einstein’s agreement for repeated indices, it is denoted: $a_{ij,j}=\sum_{j}^{3}\left(\frac{\partial a_{ij}}{\partial x_{j}}\right)$.

Considering Equation (2) and assumption ${\left(f_{\rho }\right)}_{,t}=0$, the moment of the force for the local volume located in the region of the phase boundary from the side of phase 1 motion has the form:

(3) $$\frac{dp^{v}}{dt}=\int_{\Omega^{v}}^{}\rho^{v}(1-f_{\rho })v_{i}{}_{,t}d\Omega .$$

and from the side of phase 2:

(4) $$\frac{dp^{w}}{dt}=\int_{\Omega^{w}}^{}\rho^{w}f_{\rho }w_{k}{}_{,t}d\Omega .$$

The law of equilibrium of the moment for a deformable medium enclosed in a volume Ω with a surface area *A* is given by the expression:

(5) $$\frac{dp_{i}{}^{v}}{dt}=\int_{A^{v}}^{}T_{i}^{v}dA+\int_{\Omega^{v}}^{}F_{\Omega_{i}}d\Omega ,$$

where $F_{\Omega }$ are the specific volumetric forces expended to change the structure of the material; and $T_{i}^{v}$ are the surface forces associated with deformation due to plastic strain and determined using the Cauchy equation:

(6) $$T_{i}^{v}=\sigma_{ij}^{v}n_{j}\;\mathrm{and}\;T_{i}^{w}=\sigma_{ij}^{w}n_{j},$$

here $\sigma_{ij}^{v}$, $\sigma_{ij}^{w}$ are the components of the stress tensor.

Transforming the surface integral in Equation (5) to the volume integral and using Equation (3), we determine the local form of the equation of motion for phase 1:

(7) $$\sigma_{ij,j}+F_{\Omega_{i}}-\rho^{v}(1-f_{\rho })v_{i,t}=0.$$

Similarly, for phase 2 we find:

(8) $$\sigma_{ij,j}^{w}-F_{\Omega_{i}}-\rho^{w}f_{\rho }w_{i}{}_{,t}=0.$$

In Equations (7) and (8) the internal friction force $F_{\Omega_{i}}$ is taken into account with different signs, according to Newton’s third law, i.e., from the side of phase 2 it is a driving force (energy is released and removed), and from the side of phase 1 it is a dissipative force that absorbs energy.

Summing Equations (7) and (8), we obtain the condition of dynamic equilibrium of the system (phase 1—interface—phase 2):

(9) $$\left\{\begin{array}{l} \sigma_{ij,j}^{v}+\sigma_{ij,j}^{w}-[\rho^{v}(1-f_{\rho })v_{i,t}-\rho^{w}f_{\rho }w_{i,t}]=0,\\ \sigma_{ij,j}^{w}-\rho^{w}f_{\rho }w_{i,t}-F_{\Omega_{i}}=0. \end{array}\right.$$

If we neglect the dynamic terms in Equation (9), then the resulting equation is the consolidation equation.

In accordance with the first law of thermodynamics, let us write out the equation of local energy equilibrium [29]:

(10) $$\dot{K}+\dot{U}=\dot{L}+\dot{Q},$$

where *K*, *U* are the kinetic and internal energy; and *L*, *Q* are the work of external forces and heat in the volume Ω bounded by surface with area *A*.

Mechanical power $\dot{L}$ is equal to the sum of surface forces, $T^{v}$ and $T^{w}$, acting on the surface *A*, volumetric (mass) forces acting inside the volume Ω:

(11) $$\dot{L}=\int_{A}^{}T^{v}(1-f_{\rho })v_{i}dA+\int_{A}^{}T^{w}f_{\rho }w_{i}dA.$$

According to the Cauchy Equation (6):

(12) $$\dot{L}=\int_{\Omega }^{}\sigma_{ij,j}^{v}(1-f_{\rho })v_{i}d\Omega +\int_{\Omega }^{}\sigma_{ij,j}^{w}f_{\rho }w_{i}d\Omega +\int_{\Omega }^{}\sigma_{ij}^{v}(1-f_{\rho })v_{i,j}d\Omega +\int_{\Omega }^{}\sigma_{ij}^{w}f_{\rho }w_{i,j}d\Omega .$$

Considering the fact that $\sigma_{ij}=\sigma_{ji}$, ${\dot{\varepsilon }}_{ij}^{v}=\frac{1}{2}(v_{i,j}+v_{j,i})$, ${\dot{\varepsilon }}_{ij}^{w}=\frac{1}{2}(w_{i,j}+w_{j,i})$, and, taking into account the equilibrium Equation (9), as well as adding the identical expression $\int_{\Omega }^{}(1-f_{\rho })[\sigma_{ij,j}^{w}\cdot v_{i}-\sigma_{ij,j}^{w}\cdot v_{i}]d\Omega \equiv 0$ to the right side of Equation (12), we obtain the power of internal forces in local form:

(13) $$\dot{L}=\sigma_{ij}^{v}{\dot{\varepsilon }}_{ij}^{v}(1-f_{\rho })+\sigma_{ij}^{w}{\dot{\varepsilon }}_{ij}^{w}f_{\rho }+F_{\Omega_{i}}[f_{\rho }w_{i}-(1-f_{\rho })v_{i}]=\rho^{v}{\left(1-f_{\rho }\right)}^{2}v_{i,t}v_{i}+\rho^{w}f_{\rho }{}^{2}w_{i,t}w_{i}.$$

Representing the absolute motion of a material particle on the interface as the sum of the displacement of phase 1 with velocity $(1-f_{\rho })v_{i}$ and displacement of phase 2 with velocity $f_{\rho }w_{i}$, we find the material derivative of kinetic energy:

(14) $$\dot{K}=\int_{\Omega }^{}[\rho^{v}{\left(1-f_{\rho }\right)}^{2}v_{i}v_{i,t}+\rho^{w}f_{\rho }{}^{2}w_{i}w_{i,t}]d\Omega .$$

Non-mechanical power $\dot{Q}$ is related to the flow of heat $\bar{q}$ through a surface as well as heat transfer through that surface. The description of this power is as follows:

(15) $$\dot{Q}=-\int_{A^{v}}^{}q_{k}n_{k}dA+\int_{A^{v}}^{}\Delta T\left[\rho^{w}c^{w}f_{\rho }w_{k}-\rho^{v}c^{v}(1-f_{\rho })v_{k}\right]n_{k}dA,$$

where *c* is the specific heat; $\Delta T=T-T_{0}$ is the temperature difference; and $T_{0}$ is the initial temperature of phase precipitation. Passing from the integrals over the surface to the integral over the volume, we obtain:

(16) $$\dot{Q}=-\int_{\Omega }^{}q_{k,k}d\Omega +\int_{\Omega }^{}\Delta T_{,k}\left[\rho^{w}c^{w}f_{\rho }w_{k}-\rho^{v}c^{v}(1-f_{\rho })v_{k}\right]d\Omega .$$

Substituting Equations (13), (14) and (16) in Equation (10) and solving it with respect to the internal energy, we obtain in the local form:

(17) $$\dot{U}=\sigma_{ij}^{v}{\dot{\varepsilon }}_{ij}^{v}(1-f_{\rho })+\sigma_{ij}^{w}{\dot{\varepsilon }}_{ij}^{w}f_{\rho }+F_{\Omega_{i}}[f_{\rho }w_{i}-(1-f_{\rho })v_{i}]-q_{i,i}-\Delta T_{,i}\left[\rho^{w}c^{w}f_{\rho }w_{i}-\rho^{v}c^{v}(1-f_{\rho })v_{i}\right].$$

Analysis of the local Equation (17) shows that the change in internal energy is caused by the work done by external forces (term in the first brackets), internal friction in the phase interface area (second term), as well as heat, conducted and transferred (last term). The concept of internal friction in the area of the interface of the phases is conditional because it is dictated by the formal similarity with the friction power, which is proportional to the speed of relative movement of the contacting surfaces at their interface.

In general, Equation (17) as a system represents a record of the first law of thermodynamics for a local volume near the interface.

The total entropy *S* in the volume Ω of the medium decomposes into the entropy ${\dot{S}}_{l}$, which is changed by the environment through the surface A bounding the volume under consideration, and the irreversibly increasing entropy *S_i_*.

The internal entropy per unit volume $S_{\mathrm{int}}$ according to the Clausius-Duhem inequality [30], which is expressed in terms of local heat transfer across the interface and local entropy S, leads to the inequality:

(18) $${\dot{S}}_{\mathrm{int}}=\dot{S}+{\left\{\frac{q_{l}}{T}-\left[\rho^{v}c^{v}(1-f_{\rho })v_{l}+\rho^{w}c^{w}f_{\rho }w_{l}\right]\frac{\Delta T}{T}\right\}}_{,l}\geq 0.$$

Differentiating the second term in Equation (18), we acquire the following expression:

(19) $${\dot{S}}_{\mathrm{int}}=\dot{S}+\left\{q_{l,l}-\left[\rho^{w}c^{w}f_{\rho }w_{l}-\rho^{v}c^{v}(1-f_{\rho })v_{l}\right]\Delta T_{,l}\right\}\frac{T}{T^{2}}-\left\{q_{l}-\left[\rho^{w}c^{w}f_{\rho }w_{l}-\rho^{v}c^{v}(1-f_{\rho })v_{l}\right]\Delta T\right\}\Delta T_{,l}\frac{1}{T^{2}}\geq 0.$$

Using the first law of thermodynamics, Equation (17), for the inequality term $\left[q_{l,l}-(\rho^{w}c^{w}f_{\rho }w_{l}-\rho^{v}c^{v}(1-f_{\rho })v_{l})\Delta T_{,l}\right]$, we rewrite the latter as follows:

(20) $${\dot{S}}_{\mathrm{int}}=\dot{S}-\frac{\dot{U}}{T}+\left\{\sigma_{ij}^{v}{\dot{\varepsilon }}_{ij}^{v}(1-f_{\rho })+\sigma_{ij}^{w}{\dot{\varepsilon }}_{ij}^{w}f_{\rho }+F_{\Omega_{i}}\left[f_{\rho }w_{i}-(1-f_{\rho })v_{i}\right]\right\}\frac{1}{T}-\left\{q_{l}-\left[\rho^{v}c^{v}(1-f_{\rho })v_{l}+\rho^{w}c^{w}f_{\rho }w_{l}\right]\Delta T\right\}\Delta T_{,l}\frac{1}{T^{2}}\geq 0.$$

Consider the Helmholtz free energy: F=U−ST and write inequality (20) as a function of the free energy (−U=−F−ST):

(21) $$-\dot{F}+\sigma_{ij}^{v}{\dot{\varepsilon }}_{ij}^{v}(1-f_{\rho })+\sigma_{ij}^{w}{\dot{\varepsilon }}_{ij}^{w}f_{\rho }+F_{\Omega_{i}}\left[f_{\rho }w_{i}-(1-f_{\rho })v_{i}\right]-\left\{q_{l}-\left[\rho^{v}c^{v}(1-f_{\rho })v_{l}+\rho^{w}c^{w}f_{\rho }w_{l}\right]\Delta T\right\}\frac{\Delta T_{,l}}{T^{2}}\geq 0.$$

We represent the free energy *F* in the form of a functional $F=(\varepsilon_{ij}^{v},\varepsilon_{ij}^{w},T,f_{\rho })$ such that there is a linear combination $F=(1-f_{\rho })F^{v}(\varepsilon_{ij}^{v},T)+f_{\rho }F^{w}(\varepsilon_{ij}^{w},T)$. In this case, the total derivative of the free energy *F* is equal to:

(22) $$\begin{array}{l} \dot{F}=(1-f_{\rho })\frac{\partial F^{v}}{\partial \varepsilon_{ij}^{v}}{\dot{\varepsilon }}_{ij}^{v}+f_{\rho }\frac{\partial F^{w}}{\partial \varepsilon_{ij}^{w}}{\dot{\varepsilon }}_{ij}^{w}+\frac{\partial F}{\partial T}\dot{T},\\ F=\int_{t}\dot{F}dt+F(0,0,T). \end{array}$$

With respect to the above defined meaning for F in Equation (22), Equation (21) can be written in the following form:

(23) $$\begin{array}{l} \left(1-f_{\rho }\right)\left(\sigma_{ij}^{v}-\frac{\partial F^{v}}{\partial \varepsilon_{ij}^{v}}\right){\dot{\varepsilon }}_{ij}^{v}+f_{\rho }\left(\sigma_{ij}^{w}-\frac{\partial F^{w}}{\partial \varepsilon_{ij}^{w}}\right){\dot{\varepsilon }}_{ij}^{w}+\frac{\partial F}{\partial T}T+F_{\Omega_{i}}{\left[f_{\rho }w_{i}-\left(1-f_{\rho }\right)v_{i}\right]}_{i}-\\ -\left\{q_{l}-\left[\rho^{w}c^{w}f_{\rho }w_{l}-\rho^{v}c^{v}\left(1-f_{\rho }\right)v_{l}\right]\Delta T\right\}\Delta T_{,l}\frac{1}{T}\geq 0,\\ \frac{\partial F}{\partial T}=-S. \end{array}$$

Obviously, the inequality in Equation (23) is always satisfied if it contains the following equalities:

(24) $$\sigma_{ij}^{v}=\frac{\partial F^{v}}{\partial \varepsilon_{ij}^{v}},\sigma_{ij}^{w}=\frac{\partial F^{w}}{\partial \varepsilon_{ij}^{w}},$$

(25) $$P_{D}=P_{D1}+P_{D2}\geq 0,$$

here

(26) $$\begin{array}{l} P_{D1}=F_{\Omega_{i}}\left[f_{\rho }w_{i}-(1-f_{\rho })v_{i}\right],\\ P_{D2}=-\left\{q_{l}-\left[\rho^{w}c^{w}f_{\rho }w_{l}-\rho^{v}c^{v}(1-f_{\rho })v_{l}\right]\Delta T\right\}\Delta T_{,l}\frac{1}{T}, \end{array}$$

where $P_{D}$ is the power dissipation function, its first term $P_{D1}$ is associated with the energy consumption for internal friction caused by the relative displacement of the phase boundaries, and the second term $P_{D2}$ is the heat transfer law.

Due to the fact that $P_{D2}\geq 0$, the first equality for $P_{D1}$ in the system of Equations (25) and (26) can be considered independently, i.e., the following is fair:

(27) $$P_{D1}=F_{\Omega_{i}}\left[f_{\rho }w_{i}-(1-f_{\rho })v_{i}\right]\geq 0.$$

The factor $f_{\rho }w_{i}-(1-f_{\rho })v_{i}$ in Equation (27) is the relative velocity of the phase displacement on their interface.

From the point of view of energy balance, it is natural to assume that $F_{\Omega_{i}}$ is the functional of internal friction between phases; therefore, regardless of the nature of the deformation, $F_{\Omega_{i}}$ should be monotonic with the rate of relative phase displacement. Considering Equation (27) and the last assumption, we have:

(28) $$F_{\Omega_{i}}=b_{i}\left[f_{\rho }w_{i}-(1-f_{\rho })v_{i}\right],$$

where *b_i_* is the positive coefficient regarding internal friction resistance.

Let us represent free energy *F* in the form:

(29) $$F(\varepsilon_{2}^{v},\varepsilon^{v},\varepsilon_{2}^{w},\varepsilon^{w},T)=(1-f_{\rho })F^{v}(\varepsilon_{2}^{v},\varepsilon^{v},T)+f_{\rho }F^{w}(\varepsilon_{2}^{w},\varepsilon^{w},T),$$

where $\varepsilon^{v}=\frac{1}{3}\varepsilon_{ij}^{v}\delta_{ij}$, $\varepsilon^{w}=\frac{1}{3}\varepsilon_{ij}^{w}\delta_{ij}$ are the volumetric strains; and $\varepsilon_{2}^{v}=\frac{\sqrt{2}}{3}\sqrt{\left(\varepsilon_{ij}^{v}-\delta_{ij}\varepsilon^{v})(\varepsilon_{ij}^{v}-\delta_{ij}\varepsilon^{v}\right)}$, $\varepsilon_{2}^{w}=\frac{\sqrt{2}}{3}\sqrt{\left(\varepsilon_{ij}^{w}-\delta_{ij}\varepsilon^{w})(\varepsilon_{ij}^{w}-\delta_{ij}\varepsilon^{w}\right)}$ are the strain intensities.

Then, we expand F into a Taylor series in the vicinity of the natural state F(0,0,0,0,T) with respect to the kinetic variables, neglecting the terms higher than the second order for the kinetic variables:

(30) $$\begin{array}{l} F=F(0,0,0,0,T)+(1-f_{\rho })\left(\frac{\partial F^{v}(0,0,T)}{\partial \varepsilon^{v}}\right)\varepsilon^{v}+(1-f_{\rho })\left(\frac{\partial F^{v}(0,0,T)}{\partial \varepsilon_{2}^{v}}\right)\varepsilon_{2}^{v}+f_{\rho }\left(\frac{\partial F^{w}(0,0,T)}{\partial \varepsilon^{w}}\right)\varepsilon^{w}+\\ +f_{\rho }\left(\frac{\partial F^{w}(0,0,T)}{\partial \varepsilon_{2}^{w}}\right)\varepsilon_{2}^{w}dt+\frac{f_{\rho }-1}{2}\left(\frac{\partial^{2}F^{v}(0,0,T)}{\partial {\left(\varepsilon^{v}\right)}^{2}}\right){\left(\varepsilon^{v}\right)}^{2}+\frac{f_{\rho }}{2}\left(\frac{\partial^{2}F^{w}(0,0,0,0,T)}{\partial {\left(\varepsilon^{w}\right)}^{2}}\right){\left(\varepsilon^{w}\right)}^{2}. \end{array}$$

Considering the formula for the Helmholtz free energy (21) and Equation (23), we acquire the constitutive equations:

(31) $$\begin{array}{l} \sigma_{ij}^{v}=\frac{2\mu^{v}}{\varepsilon_{2}^{v}}(\varepsilon_{ij}^{v}-\delta_{ij}\varepsilon^{v})+\left(H^{v}\varepsilon^{v}+\frac{\partial F^{v}(0,0,T)}{\partial \varepsilon^{v}}+\sigma^{v}\right)\delta_{ij},\\ \sigma_{ij}^{w}=\frac{2\mu^{w}}{\varepsilon_{2}^{w}}(\varepsilon_{ij}^{w}-\delta_{ij}\varepsilon^{w})+\left(H^{w}\varepsilon^{w}+\frac{\partial F^{w}(0,0,T)}{\partial \varepsilon^{w}}+\sigma^{w}\right)\delta_{ij}, \end{array}$$

here $\sigma^{v}=\frac{1}{3}\sigma_{ij}^{v}\delta_{ij}$, $\sigma^{w}=\frac{1}{3}\sigma_{ij}^{w}\delta_{ij}$ are the hydrostatic pressure; $\mu^{w}=\left(\frac{\partial F^{v}(0,0,T)}{\partial \varepsilon_{2}^{v}}\right)$, $\mu^{w}=\left(\frac{\partial F^{w}(0,0,T)}{\partial \varepsilon_{2}^{w}}\right)$ are the shear modulus for phase 1 and 2; and $H^{v}=\frac{\partial^{2}F^{v}(0,0,T)}{{\left(\partial \varepsilon^{v}\right)}^{2}}$, $H^{w}=\frac{\partial^{2}F^{w}(0,0,T)}{{\left(\partial \varepsilon^{w}\right)}^{2}}$ are the bulk modulus of phase 1 and 2.

To define $\frac{\partial F^{v}}{\partial \varepsilon^{v}}$ and $\frac{\partial F^{w}}{\partial \varepsilon^{w}}$ in Equation (31), let us imagine that the system has the possibility of stress-free thermal expansions. In this case, in view of the absence of shear strains ($\varepsilon_{ij}^{v}=\varepsilon_{ij}^{w}=0$ for *i* ≠ *j*) and considering that $\varepsilon^{w}=\alpha_{m}^{w}\Delta T$, $\varepsilon^{v}=\alpha_{m}^{v}\Delta T$ are the thermal deformation, from Equation (31) we obtain:

(32) $$\begin{array}{l} -\frac{\partial F^{w}(0,0,T)}{\partial \varepsilon^{w}}=v_{m}^{w}\Delta T,\\ -\frac{\partial F^{v}(0,0,T)}{\partial \varepsilon^{v}}=v_{m}^{v}\Delta T, \end{array}$$

where $v_{m}^{v}=H^{v}\alpha_{m}^{v}$, $v_{m}^{w}=H^{w}\alpha_{m}^{w}$; $\alpha_{m}^{v}$, $\alpha_{m}^{w}$ are the volumetric coefficients of thermal expansion.

Having identified the physical meaning of all the coefficients in Equation (31), we write down the constitutive equations connecting the components of the stress and strain tensors:

(33) $$\begin{array}{l} \sigma_{ij}^{v}=\frac{2\mu^{v}}{\varepsilon_{2}^{v}}\varepsilon_{ij}^{v}+(H^{v}\varepsilon^{v}-v_{m}^{v}\Delta T+\sigma^{v})\delta_{ij},\\ \sigma_{ij}^{w}=\frac{2\mu^{w}}{\varepsilon_{2}^{w}}\varepsilon_{ij}^{w}+(H^{w}\varepsilon^{w}-v_{m}^{w}\Delta T+\sigma^{w})\delta_{ij}. \end{array}$$

Let us determine the initial term F(0,…,0,T) of Equation (30), for which we use the relation known in thermodynamics:

(34) $$c_{\Omega }=c_{\varepsilon^{w},\varepsilon^{v}}=T{\left(\frac{\partial S}{\partial T}\right)}_{\varepsilon^{w},\varepsilon^{v}}=-T{\left(\frac{\partial^{2}F}{\partial T^{2}}\right)}_{\varepsilon^{w},\varepsilon^{v}},$$

where $c_{\Omega }$ is the isochoric heat capacity of the medium.

Integration of the dependence for $c_{\Omega }$ in Equation (34) over temperature *T* leads to the estimate of F(0,…,0,T):

(35) $$F(0,\ldots ,0,T)=-\int_{T_{0}}^{T}dT\int_{T_{0}}^{T}\frac{c_{\Omega }}{T}dT.$$

Now we can write down all the entered coefficients of the state function, Equation (30), in an explicit form, considering Equations (33) and (35):

(36) $$F=F_{def}(\varepsilon_{ij}^{v},\varepsilon_{ij}^{w},\varepsilon^{v},\varepsilon^{w})-3\left[(1-f_{\rho })\varepsilon^{v}v_{m}^{v}+f_{\rho }\varepsilon^{w}v_{m}^{w}\right]\Delta T-\int_{T_{0}}^{T}dT\int_{T_{0}}^{T}\frac{c_{\Omega }}{T}dT,$$

here

(37) $$F_{def}=\frac{3}{\sqrt{2}}(1-f_{\rho })\mu^{v}\varepsilon_{2}^{v}+f_{\rho }\mu^{w}\varepsilon_{2}^{w}+\frac{f_{\rho }}{2}H^{w}{\left(\varepsilon^{w}\right)}^{2}+\frac{1-f_{\rho }}{2}H^{v}{\left(\varepsilon^{v}\right)}^{2}.$$

As a result, considering the dependence $\varepsilon_{m}{}^{v}=\alpha_{m}^{v}\Delta T$, where $\varepsilon_{m}^{v}=\frac{1}{3}{\left(\varepsilon_{m}^{v}\right)}_{i}\delta_{i}$, $\alpha_{m}^{v}=3{\left(\alpha_{m}^{v}\right)}_{i}$, we obtain:

(38) $$F=F_{def}(\varepsilon^{v},\varepsilon^{w},\varepsilon_{2}^{v},\varepsilon_{2}^{w})-\frac{3}{2}(1-f_{\rho })H^{v}{\left(\alpha_{m}^{v}\Delta T\right)}^{2}-\frac{3}{2}f_{\rho }H^{w}{\left(\alpha_{m}^{w}\Delta T\right)}^{2}-\int_{T_{0}}^{T}dT\int_{T_{0}}^{T}\frac{c_{\Omega }}{T}dT.$$

In order to obtain the heat conduction equation, we introduce the Gibbs function *G*, defined through the work of deformation:

(39) $$G=F-(1-f_{\rho })\sigma_{ij}^{v}\varepsilon_{ij}^{v}-f_{\rho }\sigma_{ij}^{w}\varepsilon_{ij}^{w}.$$

We define *G* in terms of stresses, for which we substitute the correlation between stress and strains, Equation (33), in Equations (38) and (39):

(40) $$G=G(\sigma_{ij}^{v},\sigma_{ij}^{w})-\frac{3}{2}(1-f_{\rho })H^{v}{\left(\alpha_{m}^{v}\Delta T\right)}^{2}-\frac{3}{2}f_{\rho }H^{w}{\left(\alpha_{m}^{w}\Delta T\right)}^{2}-\int_{T_{0}}^{T}dT\int_{T_{0}}^{T}\frac{c_{\Omega }}{T}dT.$$

Let us express the isobaric heat capacity *c_p_* in terms of the Gibbs energy *G*:

(41) $$c_{p}=c_{\sigma }=T{\left(\frac{\partial S}{\partial T}\right)}_{\sigma_{ij}^{v},\sigma_{ij}^{w}}=-T{\left(\frac{\partial^{2}G}{\partial T^{2}}\right)}_{\sigma_{ij}^{v},\sigma_{ij}^{w}}.$$

Differentiating Equation (41) considering the found value for *G* in Equation (40), we obtain the differences in specific heat at constant pressure and at constant volume:

(42) $$c_{\sigma }^{}-c_{\Omega }^{}=3(1-f_{\rho })H^{v}{\left(\alpha_{m}^{v}\right)}^{2}T+3f_{\rho }H^{w}{\left(\alpha_{m}^{w}\right)}^{2}T.$$

Assuming $c_{\sigma }^{}-c_{\Omega }^{}=(1-f_{\rho })(c_{\sigma }^{v}-c_{\Omega }^{v})+f_{\rho }(c_{\sigma }^{w}-c_{\Omega }^{w})$, for our case we obtain:

(43) $$\begin{array}{l} c_{\sigma }^{v}-c_{\Omega }^{v}=3H^{v}{\left(\alpha_{m}^{v}\right)}^{2}T,\\ c_{\sigma }^{w}-c_{\Omega }^{w}=3H^{w}{\left(\alpha_{m}^{w}\right)}^{2}T, \end{array}$$

where:

(44) $$\begin{array}{l} v_{m}^{v}=\frac{c_{\sigma }^{v}-c_{\Omega }^{v}}{3\alpha_{m}^{v}T},\\ v_{m}^{w}=\frac{c_{\sigma }^{w}-c_{\Omega }^{w}}{3\alpha_{m}^{w}T}. \end{array}$$

Let us calculate the material derivative of the internal energy (U=F−TS), where free energy *F* is given by Equations (36) and (37). Considering the constitutive Equation (33), we have:

(45) $$\dot{U}=\left[(1-f_{\rho })\sigma_{ij}^{v}{\dot{\varepsilon }}_{ij}^{v}+f_{\rho }\sigma_{ij}^{w}{\dot{\varepsilon }}_{ij}^{w}\right]+\left\{\left[(1-f_{\rho })v_{m}^{v}{\dot{\varepsilon }}^{v}+f_{\rho }v_{m}^{w}{\dot{\varepsilon }}^{w}\right]T+c_{\Omega }\dot{T}\right\}.$$

According to Equation (36), the second term in Equation (45) is equal to the product of the material derivative of entropy and temperature:

(46) $$T\dot{S}=\left[(1-f_{\rho })v_{m}^{v}{\dot{\varepsilon }}^{v}+f_{\rho }v_{m}^{w}{\dot{\varepsilon }}^{w}\right]T+c_{\Omega }\dot{T}.$$

Comparing the expressions for the material derivative of internal energy $\dot{U}$ in the form (45) and (17), we obtain:

(47) $$T\dot{S}=3\left[(1-f_{\rho })v_{m}^{v}{\dot{\varepsilon }}^{v}+f_{\rho }v_{m}^{w}{\dot{\varepsilon }}^{w}\right]T+c_{\Omega }\dot{T}=F_{\Omega }\left[f_{\rho }w_{i}-(1-f_{\rho })v_{i}\right]-q_{i,i}+\Delta T_{,i}\left[\rho^{w}c^{w}f_{\rho }w_{i}-\rho^{v}c^{v}(1-f_{\rho })v_{i}\right].$$

Substituting into Equation (47) the heat transfer law

(48) $$q_{l}=-\lambda \Delta T_{,l}+\left[\rho^{w}c^{w}f_{\rho }w_{l}-\rho^{v}c^{v}(1-f_{\rho })v_{l}\right]\Delta T,$$

the medium flow law, Equation (28), and the differences in specific heat, Equation (42), we obtain the equation for heat transfer:

(49) $$\lambda \nabla^{2}\Delta T+b_{i}\left[f_{\rho }w_{i}-(1-f_{\rho })v_{i}\right]=c_{\Omega }\Delta \dot{T}+f_{\rho }\frac{c_{\sigma }^{w}-c_{{}_{\Omega }}^{w}}{\alpha_{m}^{w}}{\dot{\varepsilon }}^{w}+(1-f_{\rho })\frac{c_{\sigma }^{v}-c_{{}_{\Omega }}^{v}}{\alpha_{m}^{v}}{\dot{\varepsilon }}^{v},$$

where *λ* is the coefficient of thermal conductivity.

Considering the dependences for an isotropic material ${\dot{\varepsilon }}^{v}=\frac{1}{3}v_{i,i}\delta_{ii}$, ${\dot{\varepsilon }}^{w}=\frac{1}{3}w_{i,i}\delta_{ii}$, $\alpha_{m}^{v}=3{\left(\alpha_{m}^{v}\right)}_{i}$, $\alpha_{m}^{w}=3{\left(\alpha_{m}^{w}\right)}_{i}$, the value of internal friction between phases in Equation (28), as well as neglecting heat transfer to the external environment $\nabla^{2}\Delta T=0$, the heat transfer Equation (49) can be rewritten

(50) $$b_{i}{\left[f_{\rho }w_{i}-(1-f_{\rho })v_{i}\right]}^{2}=c_{\Omega }\Delta \dot{T}+f_{\rho }\frac{c_{\sigma }^{w}-c_{{}_{\Omega }}^{w}}{{\left(\alpha_{m}^{w}\right)}_{i}}w_{i,i}+(1-f_{\rho })\frac{c_{\sigma }^{v}-c_{{}_{\Omega }}^{v}}{{\left(\alpha_{m}^{v}\right)}_{i}}v_{i,i}.$$

Consolidation of the two-phase area is understood as the state of the medium without destruction of the interface zone. In this case, the condition of equality of the velocities and accelerations of material points on both sides of the interface region is fulfilled

(51) $$v_{i}=w_{i},\;v_{i,i}=w_{i,i}.$$

Equation (50) considering Equation (51) can be written as

(52) $$\left[f_{\rho }\frac{c_{\sigma }^{w}-c_{{}_{\Omega }}^{w}}{{\left(\alpha_{m}^{w}\right)}_{i}}+(1-f_{\rho })\frac{c_{\sigma }^{v}-c_{{}_{\Omega }}^{v}}{{\left(\alpha_{m}^{v}\right)}_{i}}\right]v_{i,i}-b_{i}v_{i}{}^{2}-c_{\Omega }\Delta \dot{T}=0$$

or by entering designations *A, B*, and *C*:

(53) $$Av_{i,i}+Bv_{i}{}^{2}+C=0.$$

Solution of the differential Equations (52) and (53) with respect to the speed $v_{i}$ of movement of phase 1 under the condition $\Delta \dot{T}<0$, C<0, B>0 is determined by the equation:

(54) $$v_{i}=\sqrt{-\frac{C}{B}}\left\{\frac{2}{1-\exp\left[2\sqrt{-\frac{C}{B}}\frac{B}{A}(x_{i}-x_{0i})\right]}-1\right\},$$

where $x_{i}>x_{0i}$, $x_{0i}=0$ is the reference point.

Let us take in Equation (52)

(55) $$\begin{array}{l} c\approx c_{\Omega },\\ c_{\Omega }=f_{\rho }c_{\Omega }^{w}+(1-f_{\rho })c_{\Omega }^{v}, \end{array}$$

which, for example, is the characteristic of a local fracture process, when the internal stresses change to a much greater extent than the change in the local volume.

Then, in Equation (54)

(56) $$\sqrt{-\frac{C}{B}}=\sqrt{\frac{f_{\rho }c_{{}_{\Omega }}^{w}+(1-f_{\rho })c_{{}_{\Omega }}^{v}}{f_{\rho }c_{\sigma }^{w}+(1-f_{\rho })c_{\sigma }^{v}}}v_{i}=\sqrt{L}v_{i},$$

(57) $$\frac{A}{B}=\left[\frac{f_{\rho }\frac{1-k^{w}}{{\left(\alpha_{m}^{w}\right)}_{i}}+(1-f_{\rho })\frac{1-k^{v}}{{\left(\alpha_{m}^{v}\right)}_{i}}\frac{c_{\sigma }^{v}}{c_{\sigma }^{w}}}{f_{\rho }+(1-f_{\rho })\frac{c_{\sigma }^{v}}{c_{\sigma }^{w}}}\right]\frac{v_{i}{}^{2}}{\Delta \dot{T}}=M\frac{v_{i}{}^{2}}{\Delta \dot{T}},$$

where

(58) $$k^{w}=\frac{c_{\Omega }^{w}}{c_{\sigma }^{w}}\;\mathrm{and}\;k^{v}=\frac{c_{\Omega }^{v}}{c_{\sigma }^{v}}$$

are the coefficients inversed to those of the polytropes.

Considering the accepted in Equations (56) and (58) designations, Equation (54) for the movement speed of the consolidated two-phase medium components will take the form:

(59) $$\begin{array}{l} v_{i}=v_{i}\sqrt{L}\left[\frac{2}{1-\exp\left(2\sqrt{L}M\Delta T\right)}-1\right],\\ \Delta T\approx \frac{x_{i}-x_{0i}}{v_{i}}\Delta \dot{T}<0. \end{array}$$

The Equations (54) and (59) will be fulfilled identically, if the following conditions are met:

(60) $$\begin{array}{l} J_{C/B}(f_{\rho })={\left(L-1\right)}^{2}\to \min,\\ J_{M}(f_{\rho })=M\to \min. \end{array}$$

The minimum of Equation (60) corresponds to the solution of the equations

(61) $$\begin{array}{l} \frac{\partial }{\partial f_{\rho }}J_{C/B}(f_{\rho })=0,\\ \frac{\partial }{\partial f_{\rho }}J_{M}(f_{\rho })=0. \end{array}$$

The solution of the first equation in Equation (61) gives the first condition for the consolidation of the two-phase system

(62) $$\frac{k^{v}}{k^{w}}=\frac{1-\frac{c_{\Omega }^{v}}{c_{\Omega }^{w}}}{1-\frac{c_{\sigma }^{v}}{c_{\sigma }^{w}}}.$$

The solution of the second equation in Equation (61) gives the second condition for the consolidation of the two-phase system

(63) $$\frac{1-k^{w}}{\alpha_{m}^{w}}=\frac{1-k^{v}}{\alpha_{m}^{v}}.$$

From Equations (62) and (63) it follows that in order to ensure the consolidation of a two-phase system and the formation of a stable adhesive bond from the point of view of thermodynamics, the following conditions have to be fulfilled:

(64) $$K_{1}=\frac{\left(1-\frac{c_{\Omega }^{v}}{c_{\Omega }^{w}}\right)k^{w}}{\left(1-\frac{c_{\sigma }^{v}}{c_{\sigma }^{w}}\right)k^{v}}\to 1,$$

(65) $$K_{2}=\frac{(1-k^{v})\alpha_{m}^{w}}{(1-k^{w})\alpha_{m}^{v}}\to 1.$$

## 4. Discussion

The obtained criteria (64) and (65) make it possible, first of all, to analyze the thermodynamic affinity of phases in their FGS interface area. Many researchers, noting the complexity of joining dissimilar materials, dwell on their differences in thermal expansion coefficients [2,31]. However, in addition to this, as this study showed, it is also necessary to consider the ratio of isobaric and isochoric heat capacities of the phases in the interface area, which, as characteristics of thermodynamic potentials: the Helmholtz free energy and Gibbs energy, determine the part of the mechanical work that can promote crack formation during solidification in the process of phases formation.

A large variety of data, such as phase diagram and solubility data, including consistent thermodynamic values of chemical potentials, enthalpies, entropies, thermal expansions, heats of transformations, and heat capacities, can be obtained from special databases. With the CALPHAD method, all the necessary calculations of the multi-component system’s thermodynamic properties can be performed.

The considered thermodynamic approach makes it possible to assess the danger of the occurrence of destructive residual stresses on a macroscopic scale. Macroscopic residual stresses (type I stresses) are balanced on the scale of the entire structure [32]. Residual stresses of type II, resulting from local, intermolecular phenomena, are averaged over the range of a pair of grains and are almost always present in a polycrystalline material due to different grain orientations and anisotropy inherent in the material of the crystal structure [33]. In [20], in particular, it is noted that in FGM area containing 79 wt.% SS304L and 21 wt.% IN625, cracks of hundreds of microns are formed at the boundary of different orientations of macrograins without visible macroscopic segregation.

Type III microscopic stresses are caused by intragranular defects in the crystal structure, such as dislocations, vacancies, or foreign atoms that distort the crystal lattice. Tensile residual stresses can have different origins, but all of them, in fact, cause inhomogeneous plastic strains [34] and, therefore, their presence at the interface can lead to the formation of cracks.

The fragments of the FBM interface area (Figure 3) from SS304L steel powder and aluminum alloy AlSi10Mg, obtained at different values of specific fusion energies at SLM, indicated an expansion of the gradient area in chemical composition due to an increase in convective forces of mass transfer with an increase in fusion energy.

**Figure 5:**
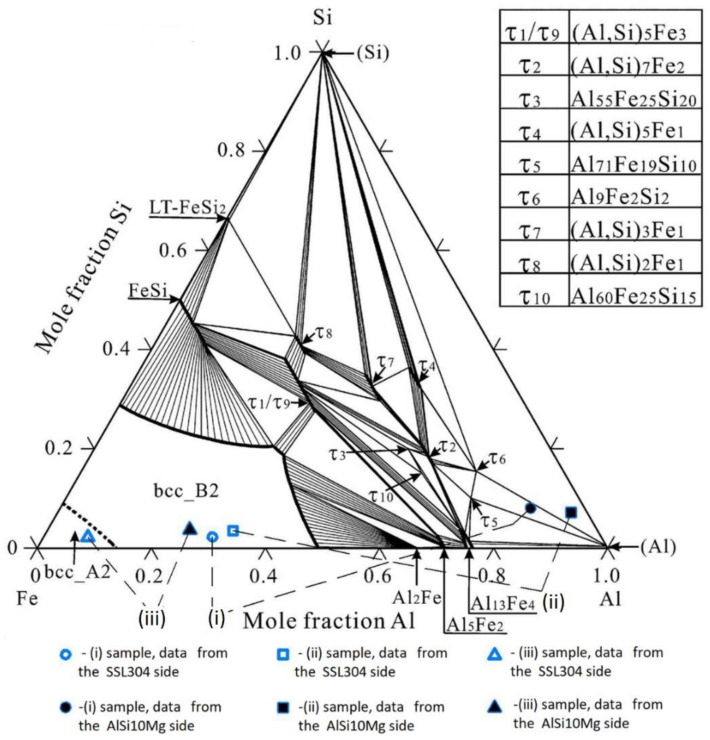
Ternary Fe-Al-Si phase equilibrium diagram at 550 °C isothermal section [35] (Reprinted with permission from Ref. [35] Elsevier 2021) with components from SS304L-AlSi10Mg interface (Figure 3); bcc_A2 and bcc_B2 are disordered and ordered BCC phases, respectively.

We took the ternary Fe-Al-Si phase equilibrium diagram at 550 °C isothermal section (Figure 5) calculated by the CALPHAD method from [35]. A multicomponent system calculation of ternary or binary systems would be required for each isothermal section and volumetric amount of the other components, but it would not add much improvement in accuracy and would provide much less clarity to the goal of the research. The Fe-Al-Si ternary phase diagram without any other component sections was chosen for the analysis due to the fact that Al has virtually unlimited solubility in Fe. The formation of intermetallics from the remaining components was not considered, since it occurs above the considered temperature of 550 °C and is limited.

In Figure 5, points corresponding to the composition of the FBM SS304L-AlSi10Mg manufactured by the SLM method at different energy densities (105, 147, 737 J/mm^3^) is also shown. With an increase in the fusion energy more than 750 J/mm^3^, the particles of aluminum powder began to evaporate, and this led to an excessive porosity and formation of cracks. Measurements of the component composition were carried out by XDR analysis at a distance of 20 μm on both sides of the interface boundary.

Analysis of the phase composition in the interface area of FBM samples showed that for the first sample (energy density was 105 J/mm^3^, Figure 3a) thermodynamic phase consolidation should be evaluated for bcc-B2 ordered structure of a solid solution based on α-Fe and phases τ_5_, τ_6_ (the amount of each phase was determined from the phase equilibrium diagram). The amount of phase τ_5_ is insignificant and it is not stable at temperatures below 610 °C [35], so it was not considered in the analysis. For the second sample (147 J/mm^3^, Figure 3b), the thermodynamic consolidation of the SS304L, phase τ_6_, and solid solution of Si in Al should be analyzed. For the third sample (737 J/mm^3^, Figure 3c) it is necessary to calculate the thermophysical characteristics of bcc-A2 disordered structure based on α-Fe and bcc-B2 ordered structure with different fractions of dissolved components.

The results of the quantitative analysis of the phase composition at the interface area of the FBM SS304L-AlSi10Mg are shown in Table 1.

From the results of the calculations the phase composition and, accordingly, the thermophysical characteristics of the phases (isochoric and isobaric heat capacities, coefficient thermal expansion), it is possible to determine the thermophysical characteristics of the material at the points under consideration. A simple rule of mixtures was used to calculate the thermophysical characteristics as a function of the material composition and temperature [36]. Modeling based on the CALPHAD method allowed us to calculate the necessary thermophysical characteristics directly under specific isothermal conditions.

**Figure 6:**
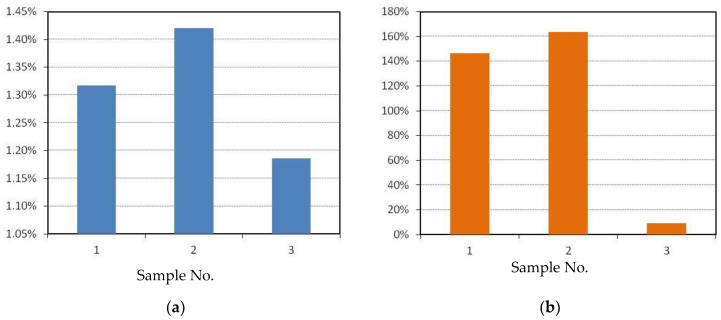
Deviations of the thermodynamic criteria of consolidation from the ideal value: (**a**) *K*_1_, (**b**) *K_2_*.

The results of calculations of the heat capacities and consolidation criteria according to Equations (64) and (65) are summarized in Table 2. Deviations of these criteria from the ideal value equal to 1 ($\left(1-K_{i}\right)/K_{i}\cdot 100\%$) are shown in Figure 6 (the smaller the deviation is, the better).

Calculated according to criteria (64) and (65), the values allowed us to estimate the thermodynamic affinity of phases for the considered samples (Figure 3) and the tendency to cracking in the interface region from the point of view of thermodynamics. The expansion of the gradient area led to a change in the consolidation criteria from the values *K*_1_ = 0.987, *K*_2_ = 0.406 for energy density 105 J/mm^3^ to *K*_1_ = 1.012, *K*_2_ = 0.916 for energy density 737 J/mm^3^, which favorably affected the resistance to cracking in the area of the layer interface from the thermodynamic point of view.

It should be noted that the considered thermodynamic consolidation does not at all guarantee the absence of cracks during solidification, since the analysis does not consider either the kinetics of phase formation or the mechanical properties of the phases.

For example, for stainless steels 304L, which was the substrate material, and 316L due to high Cr content (Cr > 20%) a hard, brittle, low-temperature equilibrium phase, σ-phase, was present. In the σ + α-phase field at temperature 475 °C, the formation of coherent chromium rich precipitates occurred within the α-matrix. These precipitates are known as α’ and form within the temperature range from 400 °C to 540 °C. The presence of the above precipitates resulted in severe embrittlement in 304L and 316L [37].

For a detailed study of the possibility of cracking, one should apply more complex models. Fracture of solids can be modelled using either a discontinuous (discrete) approach or a continuous one. In the former, the displacement field is allowed to be discontinuous across the fracture surfaces whereas in the latter the displacements are continuous everywhere but the stresses are gradually reduced to model the degradation process using some softening material models [38]. Discrete approaches using a finite element crack representation often make use of extended finite elements [39,40] and embedded discontinuities [41,42]. Strong discontinuities are embedded at the intra-element level and often require additional criteria based on, e.g., stresses, strain energy densities or energy release rates, or other special treatments and special remeshing techniques.

Difficulties associated with the discontinuous (discrete) crack modelling motivate other computational techniques in which crack paths are automatically determined as part of the solution. Two popular models falling within this category are the phase-field (PF) fracture/damage model [43] and the Peridynamics (PD), proposed by Silling [44].

In PF models, the order parameter or function that couples the order parameter is used to differentiate between broken and undamaged material, and the entire crack evolution is obtained implicitly by solving the evolution equation of the order parameter which is coupled to the mechanical field equations [45]. The meaning of auxiliary parameters, e.g., in degradation functions [46,47], is differentiating between material phases and the coupling of such parameters to continuum equations in order to obtain the characteristic self-organizing model properties.

PD is a non-local type of continuum mechanics formulation. In other words, it is a continuum formulation rather than a numerical approach in general. The governing equations of PD are integro-differential equations and do not contain spatial derivatives, which makes this new theory very attractive for problems including discontinuities such as cracks. In practice, numerical techniques are used for the solution process. The most common approach for spatial discretization is meshless scheme. Finite element discretization is also possible and available in the literature [47].

So, key advantages for both PD and PF approaches [48] are:

(i) There is no need of any initial crack, e.g., prescribed defects, in the model and cracks and fractures are initiated over time.
(ii) Notion of damage in the model representation techniques requires an additional criterion. However, in PD and PF models the criteria for the crack growth is determined as a part of the solution and no external criteria is needed.
(iii) Variational phase field methods for fracture are enjoying a notable success [49,50].

Among other applications, PF [51,52,53] and PD [54] approaches are used for FGMs that are the subject of this study.

These detailed methods introduce additional complexity when embedding models in the finite element framework, although they greatly simplify the amount of computation compared with discrete methods that require permanent remeshing. Remeshing in this case is a technique that automatically rebuilds the geometry near crack with a more uniform topology of finite element.

If we refer to the framework for the application of the obtained consolidation criteria (64) and (65), then in the opinion of the authors it looks as follows:

(i) based on the calculation of the diagrams of phase equilibrium and thermophysical properties of a multicomponent composition by the CALPHAD method using consolidation criteria, a conclusion is made about the potential possibility of crack formation, and the places of their possible formation are indicated;
(ii) if necessary, using PF and PD methods, a more detailed analysis of crack formation and spread is carried out.

We divide the main studies in this area into two groups. The first group is focused on the phase composition analysis in the region of cracks formation in terms of phase equilibrium stability or the possibility of isolating the brittle phase, which may be the cause of cracks, e.g., [13,14,15,20,21]. This approach can be referred to as the field of qualitative analysis. The second group is based on computational methods such as the phase-field method, which provides the crack development analysis, considering the energy degradation function or other criteria [39,40,41,42,43,44,45,46,47]. Quantitative estimates for these cases are obtained by numerical analysis methods. The proposed method for calculating the consolidation criteria in this study, in the difference to the noted approaches, is intended only for determining the places where cracks can potentially appear. Further, if it is necessary and such places are identified, a more detailed analysis using Finite Element (FE) methods is possible. This approach significantly reduces the search for areas for time-consuming detailed calculations.

## 5. Conclusions

The criteria for assessing the possibility of formation of strong discontinuities in the structure (cracks) in the contact boundary of the phases of a multicomponent system in the form of Equations (64) and (65) representing the thermodynamic conditions for the consolidation of two phases were proposed.

In deriving the equations of consolidation of two phases, the problem of discreteness at the phase boundary of the velocity break was solved using the Heaviside function (1), which can be approximated by a differentiable analytical dependence. The condition of consolidation (9) is considered primarily as a balance of forces between phases, which is akin to the PD approach. In the original formulation of Silling, the interaction forces between material points were assumed to be equal in magnitude and opposite to each other [42]. Approximation of the conditions of consolidation of the first order (28) made it possible to obtain the equation of heat transfer (49) as an equation of the balance of energies considering free energy as a function of the difference between isobaric and isochoric heat capacities for each phase. Additional kinematic constraints on the equality of velocities of material points on both sides of the phase interface made it possible to obtain criteria for consolidation in a simple form.

Thermophysical characteristics included in the criteria as well as the phase composition of a multicomponent structure can be obtained by the CALPHAD method by analyzing the conditions of phase equilibrium. An example of such an analysis is given in the study of the interface of the bimetallic compound SS340L-AlSi10Mg obtained by the SLM method.

So, we consider the following results:

(i) we obtained the conditions (criteria) for consolidation of multiphase material near the interface based on the difference in the chemical composition of phases using the analysis of energy in the interface region by the thermodynamics of nonequilibrium processes;
(ii) the proposed criteria were tested by analyzing the interface strength of the bi-metallic compound 304L-AlSi10Mg, which was manufactured by SLM at various energy densities of fusion;
(iii) the proposed criteria assisted the area’s indication of possible crack formation for further, more accurate analysis, for example, by using the FE method.

The proposed conditions of thermodynamic consolidation do not provide a guarantee of the absence of cracks, since the analysis does not consider either the kinetics of phase formation or the mechanical properties of the phases. The obtained criteria should be considered as indicators of the possibility of formation of strong discontinuities in the structure at phase boundaries. For a further study of such zones, other methods, such as phase-field approach or Peridynamic approach, should be used.

## Figures and Tables

**Table 1:** The phase composition at the interface boundary of FBM SS304L-AlSi10Mg.

Location	Phase Content, %		
	$\tau_{6}({\mathrm{Al}}_{9}{\mathrm{Fe}}_{2}{\mathrm{Si}}_{2})$	(Al)+(Si)	α-Fe(bcc)
Sample 1 (105 J/mm^3^)			
From the substrate side	22.39	11.14	66.47
From the AlSi10Mg side	19.16	80.84	-
Sample 2 (147 J/mm^3^)			
From the substrate side	22.25	2.27	75.48
From the AlSi10Mg side	20.55	-	79.45
Sample 3 (737 J/mm^3^)			
From the substrate side	9.54	-	90.46
From the AlSi10Mg side	10.11	12.63	72.26

**Table 2:** Criteria for consolidation at the interface.

Location	Heat Capacity, J/(mol·K)			Criteria	
	$c_{\Omega }$	$c_{\sigma }$	$c_{\Omega }/c_{\sigma }$	$K_{1}$	$K_{2}$
Sample 1 (105 J/mm^3^)					
From the substrate side	237.5	244.6	0.971	0.987	0.406
From the AlSi10Mg side	81.5	86.3	0.945		
Sample 2 (147 J/mm^3^)					
From the substrate side	256.9	264.3	0.972	0.986	0.379
From the AlSi10Mg side	85.6	90.6	0.945		
Sample 3 (737 J/mm^3^)					
From the substrate side	252.2	257.6	0.979	1.012	0.916
From the AlSi10Mg side	224.9	230.0	0.978		

## Data Availability

Not applicable.

## Abbreviations

FG	Functional Graded;
FGM	Functional Graded Material;
FBM	Functional Bimetallic Material;
FGS	Functional Graded Structure;
DED	Direct Energy Deposition;
SLM	Selective Laser Melting;
FE	Finite Element;
PF method	Phase Field method;
PD method	Peridynamics method.
